# Relationship of cumulative exposure to the triglyceride-glucose index with ischemic stroke: a 9-year prospective study in the Kailuan cohort

**DOI:** 10.1186/s12933-022-01510-y

**Published:** 2022-05-03

**Authors:** Xianxuan Wang, Baoyu Feng, Zegui Huang, Zefeng Cai, Xinran Yu, Zekai Chen, Zhiwei Cai, Guanzhi Chen, Shouling Wu, Youren Chen

**Affiliations:** 1 Shantou University Medical College, Shantou, Guangdong China; 2 Department of Cardiology, Second Affiliated Hospital of Shantou University Medical College, Shantou, Guangdong China; 3 Department of Epidemiology and Biostatistics, Institute of Basic Medical Sciences Chinese Academy of Medical Sciences; School of Basic Medicine Peking Union Medical College, Beijing, China; 4 North China University of Science and Technology, Tangshan, China; 5 Department of Epidemiology, University Medical Center Groningen, University of Groningen, Groningen, Netherlands; 6 China Medical University, Shenyang, China; 7 Department of Cardiology, Kailuan General Hospital, Tangshan, China

**Keywords:** Triglyceride-glucose index, Cumulative exposure, Ischemic stroke

## Abstract

**Background:**

A single measurement of the triglyceride-glucose (TyG) index, a simple and reliable surrogate marker of insulin resistance, is associated with ischemic stroke. However, evidence for an effect of a long-term elevation in TyG index on ischemic stroke is limited. Therefore, we evaluated the relationship between cumulative TyG index exposure and the risk of ischemic stroke.

**Methods:**

A total of 54,098 participants in the Kailuan study who had not experienced ischemic stroke underwent three measurements of fasting blood glucose and triglycerides during 2006–2007, 2008–2009, and 2010–2011. Cumulative exposure to TyG index was calculated as the weighted sum of the mean TyG index value for each time interval (value × time). Participants were placed into four groups according to the quartile of the weighted mean: Q1 group, < 32.01; Q2 group, 32.01–34.45; Q3 group, 34.45–37.47; and Q4 group, ≥ 37.47. Cox proportional hazard models were used to assess the relationships of the cumulative TyG index with incident ischemic stroke by calculating hazard ratios (HRs) and 95% confidence intervals (95% CIs).

**Results:**

There were 2083 incident ischemic stroke events over the 9 years of follow-up. The risk of ischemic stroke increased with the quartile of cumulative TyG index. After adjustment for multiple potential confounders, participants in groups Q4, Q3, and Q2 had significantly higher risks of ischemic stroke, with HRs (95% CIs) of 1.30 (1.12–1.52), 1.26 (1.09–1.45), and 1.09 (0.94–1.27), respectively (*P*_*trend*_ < 0.05), compared with the Q1 group. The longer duration of high TyG index exposure was significantly associated with increased ischemic stroke.

**Conclusions:**

High cumulative TyG index is associated with a higher risk of ischemic stroke. This finding implies that monitoring and the maintenance of an appropriate TyG index may be useful for the prevention of ischemic stroke.

**Supplementary Information:**

The online version contains supplementary material available at 10.1186/s12933-022-01510-y.

## Background

Stroke is the second leading cause of death and the third leading cause of disability worldwide [1], and it has been reported that approximately 11.6% of global mortality is attributable to stroke, with approximately 6.55 million deaths owing to stroke in 2019 [2]. Ischemic stroke is the most common subtype of pathological stroke, accounting for 85% of all strokes [3]. Therefore, the prevention of stroke, and especially ischemic stroke, through better understanding and the reduction of risk factors, has significant implications for public health and clinical practice.

Insulin resistance (IR) is not only an important contributor to the progression of myocardial infarction, arterial stiffness, and metabolic syndrome [4–6], but is also an independent risk factor for ischemic stroke [7]. Therefore, the early identification and control of IR may contribute to the prevention of ischemic stroke. The triglyceride-glucose (TyG) index, which is calculated using fasting blood glucose (FBG) and fasting triglyceride (TG) concentrations, has been reported to be a reliable and simple surrogate marker of IR [8, 9], and recent cohort studies have demonstrated that an increase in TyG index is a risk factor for ischemic stroke [10, 11].

Most previous studies of the relationship between the TyG index and ischemic stroke used single measurements, but the TyG index is affected by many biological and pathological factors, such as age, diet, and medication [12]. Therefore, the relationship between cumulative TyG index and incident ischemic stroke remains to be fully characterized. In the present study, we aimed to evaluate the relationship of cumulative TyG index, which incorporates both the TyG index value and the duration of exposure to a high TyG index, with ischemic stroke using a large community-based prospective cohort derived from the Kailuan Study.

## Methods

### Study population

The Kailuan Study is an ongoing prospective community-based cohort study to investigate the risk factors for cardiovascular diseases, cerebrovascular diseases and other non-communicable diseases, which has been described in detail elsewhere [13, 14]. In briefly, the Kailuan Study was designed and initiated in 2006–2007 and a total of 101,510 participants were enrolled into participate the baseline surveys and the follow-up visits biennially. Up to now, the Kailuan cohort has completed seven circles of health assessments, including health assessments in 2006–2007, 2008–2009, 2010–2011, 2012–2013, 2014–2015, 2016–2017, and 2018–2019. According to standardized uniform design, face-to-face questionnaire interviews (demographic characteristics, disease history, lifestyles, etc.), physical examinations (body weight, height, waist circumference, blood pressure, etc.), and laboratory tests (fasting blood glucose, lipids profile, etc.) were conducted by trained physicians or nurses in every circle. The study was approved by the ethics committee of Kailuan Hospital. Written informed consent was obtained from all participants before every survey circle.

**Fig 1:**
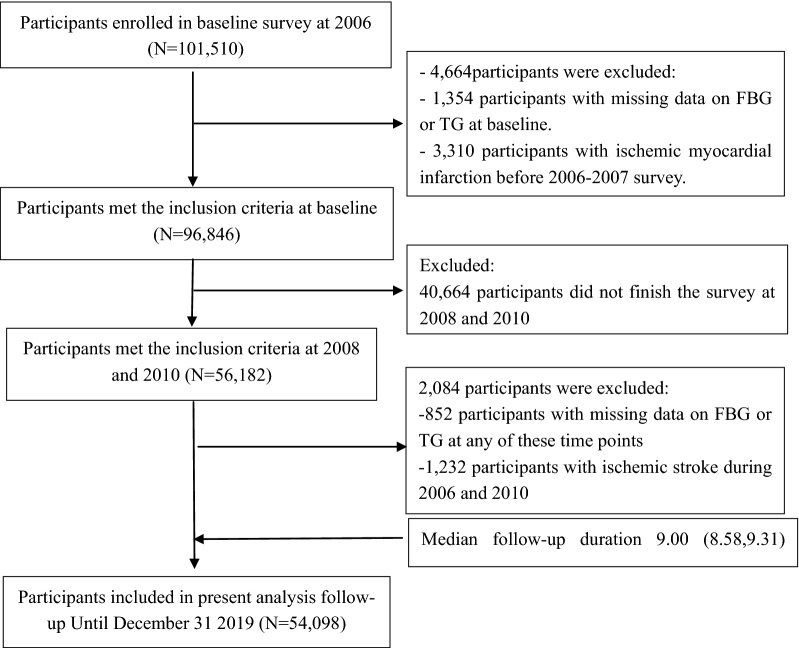
Flow chart for the inclusion of participants in the study

The present study was based on the Kailuan Study. Participants were included in the study if they had participated in the first three circles of physical examinations. The survey in 2010–2011 was regarded as the index year, the start time-point of follow-up. After excluding participants with a history of ischemic stroke prior to the third physical examination (2010–2011), or missing data on FBG or TG at each examination, a total of 54,098 participants were included for analysis (Fig. 1).

### Definition of the TyG index

The TyG index was calculated as ln [TG (mg/dL) × FBG (mg/dL)/2] [13]. Cumulative exposure to TyG index (cum-TyG) was calculated as the weighted sum of the mean TyG value for each visit: (TyG index_2006_ + TyG index_2008_)/2 × time_1–2_ + (TyG index_2008_ + TyG index_2010_)/2 × time_2–3_, where TyG index_2006_, TyG index_2008_, and TyG index_2010_ represent the TyG index at the first, second, and third examinations, and time_1–2_ and time_2–3_ represent the participant-specific time intervals between consecutive examinations (in years) [14]. The mean values of time_1–2_ and time_2–3_ were 2.07 and 1.97 years. We then placed the participants into four groups according to the quartile of cum-TyG: Q1 group, < 32.01; Q2 group, 32.01–34.45; Q3 group, 34.45–37.47; and Q4 group, ≥ 37.47.

Previous studies have shown that participants with a high TyG index are at a higher risk of ischemic stroke [15]. In the present analysis, a high TyG index was defined as a TyG index higher than the appropriate cut-off value, which was determined using a time-dependent receiver operating characteristic (ROC) curve (Additional file 1: Table S5). The duration of exposure to the TyG index was defined as the period of time during the study period in which a participant had a high TyG index: 0 years (TyG index less than the cut-off value at all three examinations), 2 years (TyG index higher than the cut-off value at one of the three examinations), 4 years (TyG index higher than the cut-off value at two of the three examinations), and 6 years (TyG index higher than the cut-off value at all three examinations).

### Outcomes

The outcome of the present study was the incidence of ischemic stroke. We used the ICD-10th revision code I63.x to identify cases of ischemic stroke [16]. Ischemic stroke was diagnosed on the basis of neurological signs, clinical symptoms, and neuroimaging, including computed tomography and magnetic resonance imaging, according to the World Health Organization criteria [17], which were consistently applied across all 11 hospitals. All the participants were followed from the index year to the first of the date of death or ischemic stroke or 31 December 2019.

### Data collection and definitions

All of the measurements were performed in a quiet, temperature-controlled room (22 °C–25 °C). All participants completed a questionnaire that collected information on their demographic characteristics (sex, age), personal health history (hypertension, diabetes, and CVD, use of antihypertensive, hyperglycemic, and lipid-lowering drugs) and lifestyle characteristics (smoking status, alcohol consumption habits, physical exercise habits) via face-to-face questionnaire interviews at each physical examination, as detailed elsewhere [18]. A current smoker was defined as someone who smoked a mean of ≥ 1 cigarette per day during the preceding year, and participants were categorized as non-smokers or current smokers. An alcohol consumer was defined as someone who drank a mean of ≥ 100 mL of alcohol per day for at least the preceding year, and participants were categorized as non-drinkers or current drinkers. Participants were categorized as undertaking physical exercise if they performed exercise ≥ 3 times per week for ≥ 30 min on each occasion [19]. Participants were asked to wear light clothes and be barefoot when measuring anthropometric indices. Body weight and height were measured to the nearest 0.1 kg and 0.1 cm, respectively, by trained physicians under standardized conditions following a standardized protocol. Body mass index (BMI) was calculated as weight (kg) divided by height squared (m^2^). Blood pressure (BP) was measured by experienced physicians using the right arm of each participant in the seated position and a calibrated mercury sphygmomanometer after 15 min of rest [20]. At least two BP measurements were made after 5 min of rest, and again if the difference between the two measurements was > 5 mmHg. The mean values were used in analyses. Hypertension [21] was defined as using a blood pressure ≥ 140/90 mmHg, the use of anti-hypertensive medication, or a self-reported history of hypertension. Diabetes [22] was defined using an FBG ≥ 7.0 mmol/L, the use of hypoglycemic drugs, or a self-reported history of diabetes. Lipid-lowering drugs were defined as drugs that lower blood lipid levels [23], such as statins, nicotinic acid, fibric acid derivatives (fibrates).

Blood samples were collected in the morning following an 8- to 12-h overnight fast at each visit. The FBG, TG, low-density lipoprotein-cholesterol (LDL-C), high-density lipoprotein-cholesterol (HDL-C), and hypersensitive C-reactive protein (hs-CRP) concentrations were measured using a Hitachi 7600 autoanalyzer (Tokyo, Japan) at the central laboratory of Kailuan General Hospital.

### Statistical analysis

Continuous, normally distributed data are summarized as mean ± standard deviation (*x̅* ± *s*) and one-way analysis of variance was used for comparisons between multiple groups. Continuous, skewed data are summarized as median and interquartile range (25%, 75%) and the Wilcoxon rank-sum test was used for comparisons between groups. Categorical variables are summarized as number and percentage (%) and the chi-square test was used for comparisons between groups. Differences of basic characteristics between four groups were compared with Bonferoni correction. The cumulative incidences of new-onset ischemic stroke for each group were calculated using the Kaplan–Meier method and these were compared using the log-rank test. Two Cox proportional hazard models were used to evaluate the relationships of the cum-TyG index and the duration of exposure to high TyG index with ischemic stroke by calculating the hazard ratios (HRs) and 95% confidence intervals (95% CIs). To reduce the effect of confounding factors, the univariate and multivariate Cox regression model was used to analyze the independent influencing factors of ischemic stroke. The variables inputted for multivariate Cox regression model were variables with a *P*-value < 0.1 (by univariate analyses). In addition, although univariate analysis results suggested that alcohol consumption was not associated with ischemic stroke, previous studies found that was strongly correlated [24]. We included the alcohol consumption in the final analysis. Data outcomes of the Cox model were listed as the HR, with a 95% CI. To assess the relationships of the cumulative TyG index, three Cox proportional hazard models were modelled with enter selection approach for covariables. In model 1, age (continuous variable, years) and sex (categorical variable, men or women) were adjusted. In Model 2, LDL-C (continuous variable, mmol/L), HDL-C (continuous variable, mmol/L), BMI (continuous variable, kg/m^2^), hs-CRP (continuous variable, mg/L), smoking status (categorical variable, smoker or non-smoker), alcohol consumption habits (categorical variable, drinker or non-drinker), physical exercise habits (categorical variable, active or inactive), hypertension (categorical variable, yes or no), diabetes mellitus (categorical variable, yes or no), and the use of lipid-lowering drugs (categorical variable, yes or no) were further adjusted. In model 3 the TyG index (continuous variable) at baseline was further adjusted. The optimum cut-off value of the TyG index for the risk of incident ischemic stroke was determined using time-dependent ROC curve analysis. The optimal cut-off value of the TyG index was identified using the maximum value of the Youden index, which was calculated as sensitivity + specificity − 1. On the whole, there were few missing data in our final analysis dataset (< 2%), and the counts and proportions of missing data for covariates are presented below. We used multiple imputation by chained equations to impute missing value for covariates [25] and the details of the missing covariates are presented in Additional file 1: Table S2.

The data were also analyzed after stratification for age and sex. To test the robustness of our findings, the following sensitivity analyses were performed: (1) the exclusion of individuals who developed ischemic stroke-related endpoints within a year (n = 381); (2) the exclusion of participants who underwent treatment with anti-hypertensive, hypoglycemic, or lipid-lowering medications (n = 11,153); (3) the exclusion of participants with abnormal FBG (≥ 7.0 mmol/L) at baseline (n = 4306); and (4) Considered that we had adjusted for hypertension in the model 3, we did not adjust for SBP. However, we found that SBP was strongly correlated with ischemic stroke in univariate analysis, we further adjusted for SBP.

We used SAS version 9.4 (SAS Institute, Cary, NC, USA) and R software (version 4.0.2) for the analyses, and a two-sided *P* value of < 0.05 was considered to represent statistical significance.

## Results

### Characteristics of the study participants

A total of 54,098 participants were included in the present study. Their mean age was 49.03 ± 11.84 years, and 76.08% were men. The baseline clinical and biochemical characteristics of the participants, categorized according to cum-TyG quartile, are shown in Table 1. Compared with Q1–Q3, participants in the Q4 tended to be older, have higher BMI, SBP, DBP, cum-TyG index, poorer metabolic profile (FBG, TG, LDL-C and hs-CRP), and with higher prevalence of hypertension and diabetes (*P* < 0.01).

**Table 1:** Baseline characteristics of participants by cumulative TyG index quartiles

	Total	Q1	Q2	Q3	Q4	*P*
Participants	54,098	13,524	13,525	13,525	13,524	
Age (years)	49.03 ± 11.84	44.88 ± 10.57	46.99 ± 11.50^a^	50.71 ± 11.89^ab^	53.54 ± 11.42^abc^	< 0.01
Male, N (%)	41,157 (76.08)	9561 (70.70)	10,782 (79.72)	10,506 (77.68)	10,308 (76.22)	< 0.01
BMI (kg/m^2^)	25.01 ± 3.16	24.03 ± 3.04	24.84 ± 3.14^a^	25.29 ± 3.10^ab^	25.87 ± 3.07^abc^	< 0.01
SBP (mmHg)	129.44 ± 16.88	123.71 ± 15.66	128.33 ± 15.64^a^	131.63 ± 16.93^ab^	134.07 ± 17.41^abc^	< 0.01
DBP (mmHg)	83.54 ± 9.22	81.19 ± 9.14	83.50 ± 8.86^a^	84.43 ± 9.21^ab^	85.06 ± 9.20^abc^	< 0.01
HDL-C (mmol/L)	1.54 ± 0.32	1.59 ± 0.32	1.55 ± 0.30^a^	1.53 ± 0.32^ab^	1.49 ± 0.33^abc^	< 0.01
LDL-C (mmol/L)	2.49 ± 0.64	2.33 ± 0.64	2.49 ± 0.60^a^	2.54 ± 0.62^ab^	2.62 ± 0.64^abc^	< 0.01
FBG (mmol/L)	5.54 ± 1.38	5.09 ± 0.68	5.35 ± 0.96^a^	5.58 ± 1.31^ab^	6.15 ± 1.99^abc^	< 0.01
TG (mmol/L)	1.34 (0.98–1.96)	0.97 (0.75–1.25)	1.32 (1.01–1.75)^a^	1.45 (1.07–2.19)^ab^	1.87 (1.37–2.72)^abc^	< 0.01
hs-CRP (mg/L)	1.45 (0.77–2.93)	1.35 (0.67–2.80)	1.34 (0.71–2.69)^a^	1.49 (0.80–2.98)^ab^	1.64 (0.92–3.19)^abc^	< 0.01
TyG index_2006_	8.64 ± 0.69	8.22 ± 0.53	8.56 ± 0.57^a^	8.74 ± 0.65^ab^	9.04 ± 0.70^abc^	< 0.01
TyG index_2008_	8.66 ± 0.68	8.19 ± 0.50	8.56 ± 0.51^a^	8.76 ± 0.61^ab^	9.15 ± 0.69^abc^	< 0.01
TyG index_2010_	8.69 ± 0.67	8.30 ± 0.51	8.61 ± 0.55^a^	8.77 ± 0.65^ab^	9.09 ± 0.70^abc^	< 0.01
Cum-TyG	34.93 ± 1.48	30.05 ± 1.62	33.23 ± 0.70^a^	35.88 ± 0.87^ab^	40.56 ± 2.61^abc^	< 0.01
Current smoking, N (%)	20,711 (38.28)	5314 (39.29)	5465 (40.41)	5111 (37.79)^ab^	4821 (35.65^)abc^	< 0.01
Current drinker, N (%)	19,167 (35.43)	4789 (35.4)	4952 (36.61)	4769 (35.26)	4657 (34.44)^ab^	< 0.01
Physical activity, N (%)	7833.0 (14.48)	1584 (11.71)	1621 (11.99)	2163 (15.99)^ab^	2465 (18.23)^abc^	< 0.01
Hypertension, N (%)	25,880 (47.84)	4649 (34.38)	6062 (44.82)^a^	7115 (52.61)^ab^	8054 (59.55)^abc^	< 0.01
Diabetes mellitus, N (%)	6022 (11.13)	470 (3.48)	930 (6.88)^a^	1605 (11.87)^ab^	3017 (22.31)^abc^	< 0.01
Antihypertensive drugs, N (%)	8397 (15.5)	1354 (10.01)	2007 (14.84)^a^	2371 (17.53)^ab^	2665 (19.71)^abc^	< 0.01
Hypoglycemic drugs, N (%)	3125 (5.78)	239 (1.77)	453 (3.35)^a^	772 (5.71)^ab^	1661 (12.3)^abc^	< 0.01
Lipid-lowering drugs, N (%)	905 (1.67)	123 (0.91)	163 (1.21)^a^	226 (1.67)^ab^	393 (2.91)^abc^	< 0.01

P, comparison of baseline characteristics between different TyG index groups

*BMI* body mass index, *SBP* systolic blood pressure, *DBP* diastolic blood pressure, *HDL-C* high-density lipoprotein cholesterol, *LDL-C* low-density lipoprotein cholesterol, *FBG* fasting blood glucose, *TG* triglyceride, *hs-CRP* high-sensitivity C reactive protein, *TyG index* triglyceride-glucose index

^a^*P* < 0.05, compare with Q1 group. ^b^*P* < 0.05, compare with Q2 group. ^c^*P* < 0.05, compare with Q3 group

### Univariate Cox regression analyses and multivariate Cox regression analyses for ischemic stroke

Univariate Cox proportional-hazards regression showed that cum-TyG index, age, gender, BMI, SBP, DBP, HDL-C, LDL-C, hs-CRP, smoking, physical exercise, taking lipid-lowering drugs, hypertension, and diabetes were significantly associated with ischemic stroke (*P* < 0.05, Table 2). Multivariate Cox proportional-hazards regression analysis revealed that cum-TyG index were independent risk factor of ischemic stroke, after adjusting for confounding factors (Table 2).

**Table 2:** Risk factors for ischemic stroke were analyzed by univariate and multivariate Cox regression analysis

	Univariate cox regression analyses		Multivariate cox regression analyses	
	HR (95% CI)	*P* value	HR (95% CI)	*P* value
Cum-TyG index	1.08 (1.07,1.10)	< 0.01	1.02 (1.01,1.03)	< 0.01
Age	1.05 (1.05,1.06)	< 0.01	1.05 (1.04,1.05)	< 0.01
Gender	2.21 (1.94,2.51)	< 0.01	1.49 (1.29,1.71)	< 0.01
BMI	1.07 (1.06,1.08)	< 0.01	1.01 (0.99,1.02)	0.49
SBP	1.04 (1.04,1.05)	< 0.01	1.02 (1.01,1.02)	< 0.01
DBP	1.06 (1.05,1.06)	< 0.01	/	/
HDL-C	0.81 (0.70,0.93)	< 0.01	0.90 (0.78,1.00)	0.06
LDL-C	1.22 (1.15,1.31)	< 0.01	1.09 (1.02,1.16)	< 0.01
hs-CRP	1.03 (1.02,1.04)	< 0.01	1.01 (1.00,1.02)	< 0.01
TyG index_2010_	1.53 (1.45,1.62)	< 0.01	1.13 (1.05,1.22)	< 0.01
Current smoking	1.30 (1.20,1.42)	< 0.01	1.42 (1.28,1.58)	< 0.01
Current drinker	1.07 (0.98,1.17)	0.12	1.00 (0.89,1.11)	0.12
Physical activity	0.81 (0.79,0.83)	< 0.01	0.89 (0.79,1.00)	0.05
Hypertension	2.65 (2.43,2.89)	< 0.01	1.02 (0.91,1.14)	< 0.01
Diabetes mellitus	2.47 (2.20,2.77)	< 0.01	1.53 (1.34,1.74)	< 0.01
Lipid-lowering drugs	0.79 (0.71,0.85)	< 0.01	0.85 (0.66,1.11)	< 0.01

### Relationship between the cumulative TyG index and risk of ischemic stroke

**Fig 2:**
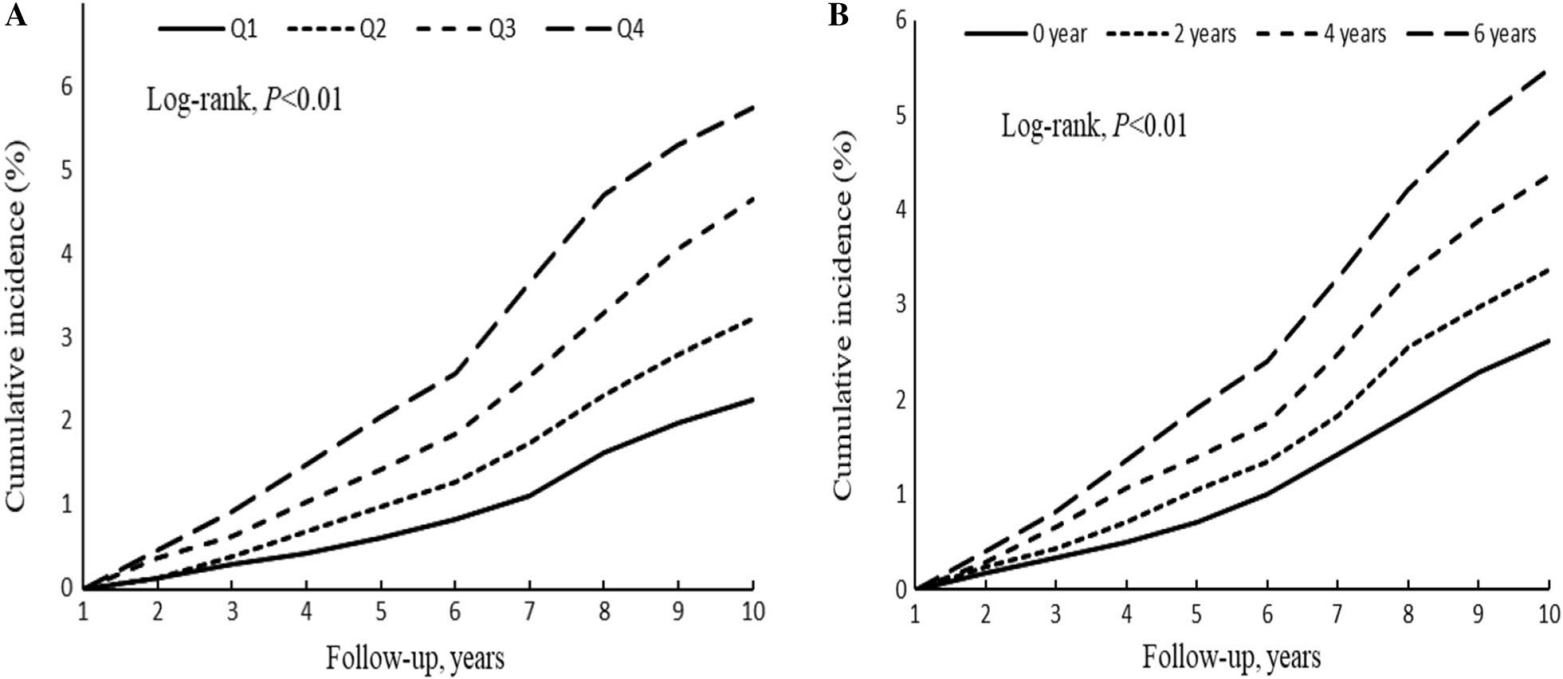
Kaplan–Meier incidence rate of ischemic stroke by TyG index. **A** Quartiles of cumulative TyG index. **B** Exposure duration with a higher TyG index

During a median follow-up period of 9 years (interquartile range 8.58, 9.31), 2083 (3.85%) participants developed ischemic stroke. The incidence rate of ischemic stroke events was 2.56, 3.66, 5.24, and 6.68 per 1000 person-years for groups Q1, Q2, Q3, and Q4 (Table 3). The 9-year cumulative incidences of ischemic stroke were 2.26%, 3.24%, 4.66%, and 5.76% for groups Q1–Q4, respectively. The cumulative incidence of ischemic stroke among groups were statistical significance calculated using the log-rank test (*P* < 0.01; Fig. 2A). After adjustment for potential confounding factors, the fully adjusted HRs (95% CIs) (Model 3) were 1.09 (95% CI 0.94–1.27), 1.26 (95% CI 1.09–1.45), and 1.30 (95% CI 1.12–1.52) for groups Q2, Q3, and Q4, respectively, compared with Q1 (Table 3).

**Table 3:** Association of cumulative TyG index with ischemic stroke

	Case/ Total	Incidence rate, per 1000 person-years	Model 1	Model 2	Model 3
Quartiles					
Q1	312/13524	2.56	1.00	1.00	1.00
Q2	435/13525	3.66	1.24 (1.07,1.44)	1.14 (0.98,1.32)	1.09 (0.94,1.27)
Q3	604/13525	5.24	1.53 (1.33,1.76)	1.34 (1.17,1.55)	1.26 (1.09,1.45)
Q4	732/13524	6.68	1.79 (1.56,2.05)	1.45 (1.25,1.67)	1.30 (1.12,1.52)
* P* for Trend			< 0.0001	< 0.0001	0.0002
Time exposure duration					
0 year	365/14630	2.89	1.00	1.00	1.00
2 years	415/12653	3.79	1.37 (1.19,1.57)	1.22 (1.06,1.40)	1.18 (1.02,1.36)
4 years	501/11733	4.95	1.77 (1.54,2.02)	1.42 (1.23,1.63)	1.32 (1.14,1.54)
6 years	802/15082	6.26	2.18 (1.92,2.47)	1.57 (1.37,1.80)	1.38 (1.16,1.64)
* P* for Trend			< 0.0001	< 0.0001	0.0003

Model 1 age (continuous variable, years) and sex (categorical variable, men or women)

Model2: included variables in model 1 and further LDL-C (continuous variable, mmol/L), HDL-C (continuous variable, mmol/L), BMI (continuous variable, kg/m^2^), hs-CRP (continuous variable, mg/L), smoking status (categorical variable, smoker or non-smoker), alcohol consumption habits (categorical variable, drinker or non-drinker), physical exercise habits (categorical variable, active or inactive), hypertension (categorical variable, yes or no), diabetes mellitus (categorical variable, yes or no), and the use of lipid-lowering drugs (categorical variable, yes or no)

Model 3: included variables in model 2 and further the TyG index (continuous variable) at baseline

### Relationship between the duration of exposure to a high TyG index and the incidence of ischemic stroke

The cut-off values determined for the prediction of ischemic stroke using TyG index were 8.66, 8.68, and 8.46 in 2006, 2008, and 2010, respectively (Additional file 1: Table S5). The incidence rate of ischemic stroke events was 2.89, 3.79, 4.95, and 6.26 per 1000 person-years for groups 0 year, 2 years, 4 years, and 6 years, respectively (Table 2). The 9-year cumulative incidences of ischemic stroke were 2.62%, 3.36%, 4.36%, and 5.48% for groups 0 year, 2 years, 4 years, and 6 years, respectively. The cumulative incidence of ischemic stroke among groups were statistical significance calculated using the log-rank test (*P* < 0.01; Fig. 2B). Participants who were found to have a high TyG index at all three visits were at a higher risk of ischemic stroke than those in the other groups. After adjustment for all the identified potential confounders, compared with the unexposed group (0 years), risk of ischemic stroke was significantly higher in those with 2 years group (HR: 1.18; 95% CI 0.95–1.25), 4 years group (HR: 1.32; 95% CI 1.14–1.54), and 6 years group (HR: 1.38; 95% CI 1.16–1.64), respectively (Table 3).

### Results of analyses of stratified data and sensitivity analyses

Additional file 1: Table. S3 shows the results of the stratified analyses. In general, a high cumulative TyG index (group Q4) and long exposure to high TyG index (6 years) were significantly associated with a higher risk of ischemic stroke across the various stratified group. The sensitivity analyses, in which individuals who developed ischemic stroke within a year; who underwent treatment with anti-hypertensive, hypoglycemic, or lipid-lowering medications; or excluded who had diabetes, or further adjusted for SBP yielded similar results (Additional file 1: Table S4).

## Discussion

In the present cohort study, we found that high cumulative TyG index was associated with a higher risk of incident ischemic stroke, independent of other conventional risk factors. In addition, longer exposures to a high TyG index were found to increase the risk of ischemic stroke. These findings were validated by studying individuals who did not have diabetes and those who were not taking anti-hypertensive, hypoglycemic, or lipid-lowering medications.

Several previous cross-sectional, prospective studies have demonstrated that the incidence of ischemic stroke is affected by many parameters, including hypertension, diabetes, hypercholesterolemia, smoking, and obesity [10, 11, 26, 27]. In this present study, we found that age, SBP, DBP, BMI, TyG index, LDL-C, hs-CRP, smoking, hypertension, diabetes were the risk factors for ischemic stroke, while HDL-C, physical exercise, and taking lipid-lowering drugs were the protect factor for ischemic stroke. What is more, after adjusted for confounding factors, we found cum-TyG index were independent risk factor of ischemic stroke. Wang et al*.* [11] found a 1.45-fold higher risk of ischemic stroke for individuals in the upper quartiles of TyG index, compared with those in the lowest quartile, but they only studied TyG measured at a single time point. In contrast, in the present study, we found that participants with a high cum-TyG index were at a higher risk of developing ischemic stroke. The risk of ischemic stroke was highest in individuals in the highest quartile group, with a multivariate-adjusted HR of 1.30 (95% CI 1.12–1.52). In addition, we found that sustained exposure to a high TyG index was a risk factor for ischemic stroke: compared with the unexposed group, a 6-year exposure to a high TyG index was associated with a 38% higher risk of ischemic stroke. In addition, the risk of ischemic stroke increased with increasing cumulative exposure to a high TyG index (*P*_*trend*_ < 0.05). The previous studies and the present findings imply that cum-TyG index exposure is an independent risk factor for ischemic stroke. Therefore, regular monitoring of the TyG index may be useful in clinical practice.

To the best of our knowledge, a few studies have examined the optimal cutoff value for TyG index for cardiovascular disease. Tian et al*.* [5] estimated TyG index cut-off value with 8.8 for cardiovascular disease, for which the area under the ROC curve was 0.751 (95% CI 0.704–0.799). However, as they did not consider the disease status changing over time, we used the time-dependent ROC to determine the optimum cut-off values of the TyG index for the risk of incident ischemic stroke, with 8.66 in 2006, 8.68 in 2008, and 8.46 in 2010, respectively. In addition, we found that the risk of ischemic stroke was lower when anti-hypertension, lipid-lowering and hypoglycemic medications were being administered [28–30]. Therefore, we repeated the analysis after the exclusion of individuals taking anti-hypertension, lipid-lowering or hypoglycemic drugs, but this did not affect the findings. Because abnormal metabolic status, including diabetes mellitus, also increases the risk of ischemic stroke [19, 31], we analyzed the data after the exclusion of participants with diabetes mellitus, and again the findings were unaffected. Thus, the association between cum-TyG index and the risk of ischemic stroke has also been demonstrated in individuals without diabetes and in those who were not taking anti-hypertensive, hypoglycemic, or lipid-lowering medications. This implies that it may be useful to monitor and maintain an appropriate TyG index over time to aid the prevention of ischemic stroke in the general population, and especially in individuals who do not have diabetes and are not taking medication.

Although we found an association between the cumulative exposure to TyG index and the risk of ischemic stroke, the underlying mechanism is unclear. However, the results of previous studies suggest that the following may be involved. First, IR increases the risks of developing chronic metabolic diseases, such as diabetes, hypertension, and dyslipidemia [32, 33], which are also risk factors for ischemic stroke. Given that individuals with IR often have multiple risk factors, their combined effect is likely to further increase the risk of ischemic stroke. Second, IR is associated with chronic inflammation, oxidative stress, and endothelial dysfunction, and accelerates the progression of atherosclerosis, which is a key component of the pathogenesis of ischemic stroke [34–37]. Finally, IR is also associated with greater platelet adhesion, activation, and aggregation, which leads to the occlusion of cerebral arteries, causing hemodynamic disturbances [38, 39].

There were several limitations to the present study. (1) It was an observational cohort study; therefore, we could not establish a causal relationship between cumulative TyG index exposure and the risk of ischemic stroke. (2) Although we adjusted for potential risk factors for ischemic stroke, other unmeasured or residual confounders, such as genetic susceptibility, may well have affected the findings. (3) We used the time-weighted method to calculate cumulative exposure to TyG index, and the time represent the participant-specific time intervals between consecutive examinations. There may slightly differ from time intervals for each person, but all participants underwent the physical examination every two years, and the means of time_1–2_ and time_2–3_ were 2.07 and 1.97 years. Therefore, differences in time internal at onset did not have a meaningful effect on the results. (4) The participants were recruited from the Kailuan Study, which is a community-based cohort study; therefore, the findings of the present study cannot be directly extrapolated to other ethnicities. However, because the population studied was quite homogeneous, the findings are likely to be reliable.

## Conclusions

In conclusion, we found that high cumulative TyG index is associated with a higher risk of incident ischemic stroke. Furthermore, long exposure to a high TyG index may increase the risk of ischemic stroke. These findings imply that monitoring and maintenance of an appropriate TyG index may be useful for the prevention of ischemic stroke.

## Supplementary Information

**Additional file 1: Table S1.** Variable assignment table for Univariate COX regression analyses and multivariate COX regression.** Table S2. **Counts and proportions of missing data. **Table S3. **Stratified analysis for the association of cumulative TyG index with ischemic stroke. **Table S4**. Sensitivity analysis for association of cumulative TyG index with ischemic stroke.** Table S5. **Receiver operative characteristics curve and cutoff value of triglyceride-glucose index for incident ischemic stroke.

## Data Availability

The datasets used and/or analyzed during the present study are available from the corresponding author on reasonable request.

## Abbreviations

BMI: Body mass index
SBP: Systolic blood pressure
DBP: Diastolic blood pressure
FBG: Fasting blood glucose
HDL-C: High-density lipoprotein-cholesterol
hsCRP: High-sensitivity C-reactive protein
IR: Insulin resistance
LDL-C: Low-density lipoprotein-cholesterol
TG: Triglycerides
TyG index: Triglyceride-glucose index
CVD: Cardiovascular disease
HR: Hazard ratio
CI: Confidence interval
ROC: Receiver operating characteristic
