# Supplementary material for: Relationship of cumulative exposure to the triglyceride-glucose index with ischemic stroke: a 9-year prospective study in the Kailuan cohort
Source: Cardiovasc Diabetol. 2022 May 3;21:66. doi: 10.1186/s12933-022-01510-y (PMC9066788; doi:10.1186/s12933-022-01510-y)
Supplement: Supplementary file 1 — Additional file 1: Table S1. Variable assignment table for Univariate COX regression analyses and multivariate COX regression. Table S2. Counts and proportions of missing data. Table S3. Stratified analysis for the association of cumulative TyG index with ischemic stroke. Table S4. Sensitivity analysis for association of cumulative TyG index with ischemic stroke. Table S5. Receiver operative characteristics curve and cutoff value of triglyceride-glucose index for incident ischemic stroke. [file 12933_2022_1510_MOESM1_ESM.docx]

**Table S1.** Variable assignment table for Univariate COX regression analyses and multivariate COX regression

| Variables |  |
| --- | --- |
| Age | Continuous variable, years |
| Body mass index | Continuous variable, kg/m^2^ |
| High-density Lipoprotein Cholesterol | Continuous variable, mmol/L |
| Low-density Lipoprotein Cholesterol | Continuous variable, mmol/L |
| High-sensitivity C Reactive Protein | Continuous variable, mg/L |
| TyG inex_2010_ | Continuous variable |
| Cum-TyG index | Continuous variable |
| Sex | 0= women, 1= men |
| Current smoking | 0= non-smoker, 1= current smoker |
| Current drinker | 0= non-drinker, 1= current drinker |
| Physical activity | 0= inactive, 1= active |
| Hypertension | 0= not, 1= yes |
| Diabetes mellitus | 0= not, 1= yes |
| Lipid-lowering drugs | 0= not, 1= yes |

**Table S2.** Counts and proportions of missing data

| Variables | Missing (%) |
| --- | --- |
| Age | 0 |
| Body mass index | 12(0.02%) |
| Systolic Blood Pressure | 1020(1.89%) |
| Diastolic Blood Pressure | 1017(1.88%) |
| High-density Lipoprotein Cholesterol | 0 |
| Low-density Lipoprotein Cholesterol | 0 |
| Fasting Blood Glucose | 0 |
| Triglyceride | 0 |
| High-sensitivity C Reactive Protein | 503(0.93%) |
| TyG inex_2006_ | 0 |
| TyG inex_2008_ | 0 |
| TyG inex_2010_ | 0 |
| Cum-TyG index | 0 |
| Current smoking | 202(0.37%) |
| Current drinker | 212(0.39%) |
| Physical activity | 205(0.38%) |
| Hypertension | 0 |
| Diabetes mellitus | 0 |
| Antihypertensive drugs | 0 |
| Hypoglycemic drugs | 0 |
| Lipid-lowering drugs | 0 |

**Table S3.** Stratified analysis for the association of cumulative TyG index with ischemic stroke

|  | Age | | Sex | |
| --- | --- | --- | --- | --- |
|  | <45 years | ≥ 45 years | Female | Male |
| **Quartiles** | | | |  |
| Q1 | 1.00 | 1.00 | 1.00 | 1.00 |
| Q2 | 1.18(0.85,1.64) | 1.09(0.92,1.29) | 1.11(0.69,1.78) | 1.09(0.93,1.27) |
| Q3 | 1.43(1.01,2.04) | 1.25(1.06,1.46) | 1.47(0.95,2.27) | 1.23(1.06,1.44) |
| Q4 | 1.82(1.24,2.67) | 1.27(1.08,1.50) | 1.61(1.03,2.51) | 1.26(1.07,1.49) |
| *P* for trend | 0.0013 | 0.0016 | 0.0162 | 0.0325 |
| **Time exposure duration** | |  |  |  |
| 0 year | 1.00 | 1.00 | 1.00 | 1.00 |
| 2 years | 1.57(1.03,2.39) | 1.11(0.95,1.30) | 1.29(0.83,2.01) | 1.14(0.98,1.33) |
| 4 years | 1.70(1.09,2.64) | 1.24(1.05,1.46) | 1.48(0.89,2.47) | 1.25(1.06,1.47) |
| 6 years | 2.00(1.23,3.26) | 1.28(1.06,1.54) | 1.69(1.08,2.64) | 1.36(1.13,1.63) |
| *P* for trend | 0.0096 | 0.0069 | 0.0002 | 0.0011 |

Model adjusted for age (continuous variable, years), sex (categorical variable, men or women), LDL-C (continuous variable, mmol/L), HDL-C (continuous variable, mmol/L), BMI (continuous variable, kg/m^2^), hs-CRP (continuous variable, mg/L), smoking status (categorical variable, smoker or non-smoker), alcohol consumption habits (categorical variable, drinker or non-drinker), physical exercise habits (categorical variable, active or inactive), hypertension (categorical variable, yes or no), diabetes mellitus (categorical variable, yes or no), the use of lipid-lowering drugs (categorical variable, yes or no), and the TyG index (continuous variable) at baseline.

**Table S4.** Sensitivity analysis for association of cumulative TyG index with ischemic stroke.

|  | Analysis 1 | Analysis 2 | Analysis 3 | Analysis 4 |
| --- | --- | --- | --- | --- |
| **Quartiles** | | | |  |
| Q1 | 1.00 | 1.00 | 1.00 | 1.00 |
| Q2 | 1.11(0.95,1.29) | 1.09(0.92,1.31) | 1.13(0.97,1.32) | 1.08(0.93,1.25) |
| Q3 | 1.23(1.06,1.43) | 1.34(1.13,1.60) | 1.26(1.10,1.49) | 1.20(1.04,1.38) |
| Q4 | 1.28(1.10,1.50) | 1.41(1.18,1.70) | 1.29(1.08,1.49) | 1.22(1.05,1.43) |
| *P* for trend | 0.0009 | <0.0001 | 0.0025 | 0.0046 |
| **Time exposure duration** | |  |  |  |
| 0 year | 1.00 | 1.00 | 1.00 | 1.00 |
| 2 years | 1.17(1.00,1.36) | 1.18(1.00,1.40) | 1.18(1.01,1.37) | 1.15(0.99,1.33) |
| 4 years | 1.32(1.13,1.54) | 1.29(1.08,1.56) | 1.30(1.07,1.57) | 1.28(1.11,1.51) |
| 6 years | 1.36(1.14,1.63) | 1.33(1.08,1.65) | 1.34(1.14,1.57) | 1.31(1.10,1.55) |
| *P* for trend | 0.0006 | 0.0075 | 0.0004 | 0.0020 |

Analysis 1: Excluding outcome events within the one year of follow-up (n=381)

Analysis 2: Excluding participants with antihypertensive, hypoglycemic, or lipid-lowering medication (n=11,153).

Analysis 3: Excluding participants with diabetes (n=4,306).

Analysis 4: Included variables in model 3 and further SBP (continuous variable, mmHg).

Model adjusted for age (continuous variable, years), sex (categorical variable, men or women), LDL-C (continuous variable, mmol/L), HDL-C (continuous variable, mmol/L), BMI (continuous variable, kg/m^2^), hs-CRP (continuous variable, mg/L), smoking status (categorical variable, smoker or non-smoker), alcohol consumption habits (categorical variable, drinker or non-drinker), physical exercise habits (categorical variable, active or inactive), hypertension (categorical variable, yes or no), diabetes mellitus (categorical variable, yes or no), the use of lipid-lowering drugs (categorical variable, yes or no), and the TyG index (continuous variable) at baseline.

**Table S5.** Receiver operative characteristics curve and cutoff value of TyG index for incident ischemic stroke.

|  | Area Under Curve | Cutoff | Sensitivity (%) | 1-Specificity (%) |
| --- | --- | --- | --- | --- |
| TyG index2006 | 0.57(0.58-0.60) | 8.66 | 60.01 | 44.10 |
| TyG index2008 | 0.58(0.57-0.59) | 8.68 | 57.82 | 44.54 |
| TyG index2010 | 0.57(0.56-0.59) | 8.46 | 75.01 | 61.13 |
